# Maternal Progesterone Level in Fetal Growth Restriction and Its Relationship with Doppler Velocimetry Indices

**Published:** 2011-03-30

**Authors:** S. Borna, M. Bandarian, A. Abdollahi, F. Bandarian, M. Malek

**Affiliations:** 1 Associate Professor, Department of Perinatology, Valiasr Hospital, Tehran University of Medical Sciences, Tehran, Iran; 2 Resident of Obstetrics and Gynecology, Valiasr Hospital, Tehran University of Medical Sciences, Tehran, Iran; 3 Pathologist, Valiasr Hospital, Tehran University of Medical Sciences, Tehran, Iran; 4 PhD Student, Endocrinology and Metabolism Research Center, Tehran University of Medical Sciences, Tehran, Iran; 5 Endocrine Research Center, Shahid Beheshti University of Medical Sciences, Tehran, Iran; 6 Imaging Center, Imam Khomeini Hospital, Tehran University of Medical Sciences, Tehran, Iran

**Keywords:** IUGR, AGA, Progesterone, Doppler

## Abstract

**Background/Objective:**

To verify whether progesterone concentration is changed in the maternal serum of intra-uterine growth retardation (IUGR) pregnancies and to assess if there is a relationship between maternal progesterone and fetal Doppler velocimetry.

**Patients and Methods:**

Thirty-five patients with intrauterine growth retardation infants and thirty-seven pregnant women with appropriate for gestational age (AGA) fetuses were enrolled in the study. Maternal progesterone serum was determined. Doppler velocimetry of umbilical and middle cerebral arteries (MCA) were obtained in all fetuses.

**Results:**

Maternal progesterone level in IUGR infants (58.49±7.06 ng/ml) had no significant difference with AGA fetuses (58.13±7.87 ng/ml) (p=0.96). In the IUGR group, umbilical artery resistive index (RI), pulsatility index (PI) and systolic/diastolic (S/D) ratio were higher than the normal group (p<0.001), and MCA RI (p value=0.014) and PI (p=0.012) were significantly less than the IUGR group. Besides, RI C/U in the IUGR group was significantly less than the normal group (p<0.001). A negative significant correlation was detected between maternal progesterone level and MCA PI (r=-0.38) and RI (r=-0.38) in the AGA group.

**Conclusion:**

It seems that progesterone has no effect on fetal placental circulation and serum progesterone can not discriminate IUGR infants from AGA infants. Progesterone is a poor marker for placental dysfunction.

## Introduction

Intra uterine growth retardation (IUGR) is failure of the fetus to reach genetic potential for growth.[1] IUGR prevalence in developed and developing countries is 4-7% and 24-30%, respectively.[2][3][4][5][6][7] IUGR is a multifactorial disorder and maternal, fetal and placental factors can interfere with normal growth mechanisms.[8]

IUGR infants are at risk for unexplained sudden intrauterine death.[9] Perinatal mortality rate among IUGR infants is higher than normal infants.[10] IUGR causes short-term morbidities like prematurity, respiratory distress syndrome and necrotizing enterocolitis along with long-term complications such as neurologic disorders, cerebral palsy and poor neurodevelopmental outcome.[9] Low birth weight is associated with coronary heart disease, stroke, hypertension and type 2 diabetes.[11]

It is commonly accepted that IUGR is associated with impairment of uteroplacental blood flow, as a result of failure of trophoblast invasion of spiral arteries, which have not been transformed to low-resistance vessels and hence blood flow is restricted in the intervillous space.[12] Therefore, fetoplacental resistance is one of the pathophysiologic mechanisms for IUGR and vasodilator deficiency may have an effect on IUGR pregnancies.

Progesterone is a smooth muscle relaxant and also has a vasodilator effect on vasculature.[13]

Ultrasound is the most accurate and sensitive method for identifying IUGR fetuses.[14] Abnormalities in terminal villous and vascular resistance in fetoplacental circulation may be assessed by Doppler velocimetry of fetal arteries and veins. During placental dysfunction, progressive vascular redistribution can be detected in fetal circulation by high umbilical artery resistance and low cerebral vascular resistance (the brain sparing effect).[15]

The aim of this study was to verify whether the progesterone level in maternal serum of IUGR pregnancies differs from normal pregnancies, and also to determine the relationship between progesterone level and Doppler velocimetry indices of the fetus.

## Patients and Methods

This case-control study was conducted among 72 pregnant women with the gestational age of 28 to 42 weeks who were referred to the perinatology clinic of Imam Khomeini Hospital for routine prenatal care and were eligible for the study. Sampling was done by census method.

IUGR was defined as fetal weight below the 10th percentile. Normal fetus was defined as fetal weight appropriate for gestational age (AGA). Thirty-five women with IUGR (case group) and 37 with a normal fetus (control group) were selected. The gestational age was determined by the first-trimester ultrasonography.

Exclusion criteria for the case group included twin pregnancy, gestational diabetes or history of diabetes mellitus, fetal anomalies, progesterone intake, and for the control group pregnancy included hypertension or preeclampsia which is also added to the previous criteria.

Informed consent was obtained from participants before enrollment and the study protocol was approved by the ethics committee of Tehran University of Medical Sciences.

All patients underwent biometric ultrasonography and Doppler velocimetry measurements. Doppler velocimetry was carried out with a commercially available instrument (L. O. GIQ500) using a 3.5-5 MHz convex probe. Doppler velocimetry consists of umbilical and middle cerebral arteries (MCA) assessment. The umbilical artery was analyzed by Pulsatility Index (PI), Resistance Index (RI), and systole to diastole ratio (S/D), while middle cerebral artery Doppler study included RI and PI. The other index was ratio of cerebral artery RI to umbilical artery RI (RI C/U). Doppler indices were calculated automatically with the software included in the ultrasound system. Maternal venous blood sample was obtained at the same day for serum progesterone assessment. Samples were centrifuged and plasma was separated and stored at -20°c until assayed.

Serum progesterone was determined by ELISA (by progesterone ELISA IBL International GmbH, Germany) intra-assay variation 5.4-6.86 (CV%) and inter-assay variation 0.96-5.59 (CV%).

Progesterone levels were adjusted according to gestational age.

Independent samples t test was applied for comparing quantitative variables with normal distribution, and Mann-Whitney U test for comparison of quantitative variables without normal distribution. Furthermore, Pearson and Spearman correlations were used to find any correlation between variables. To remove the effect of confounding factors, the multiple logistic regression model was used. P value of equal or less than 0.05 was considered statistically significant. SPSS 15 for Windows (SPSS Inc., Chicago, Illinois) was used for statistical analysis.

## Results

IUGR and control groups had the same maternal age, gravity, and gestational age. As expected, in the IUGR group, the estimated fetal weight and maternal body mass index (BMI) were significantly less than the AGA group (Table 1).

**Table 1:** Baseline Characteristics of Case and Control Groups

**Variable**	**IUGR Group (mean±SD)**	**Control Group (mean±SD)**	**P Value**
Maternal Age	29.17±6.06	28.51±3.85	0.58
Gestational Age	33.51±3.51	33.89±2.97	0.62
Parity	2.17±1.40	2.35±1.35	0.58
Estimated Fetal Weight	1614.68±493.71	2270.51±786.44	0.002^a^
Body Mass Index	23.43±3.86	26.70±4.42	0.003^a^

^a^ p≤0.05 Statistically Significant

Maternal progesterone in IUGR group (58.5±7.1) and AGA group (58.1±7.9) was not significantly different (p=0.96).

In IUGR group, umbilical artery RI, PI, and S/D were higher than those in the normal group, and MCA RI and PI were significantly less than those in the IUGR group. Besides, RI C/U in IUGR group was significantly less than that in the normal group (Table 2).

**Table 2:** Doppler Indices in IUGR and Normal Groups

**Doppler Indexes**	**IUGR Group (mean±SD)**	**Control Group (mean±SD)**	**P Value**
Umbilical Artery RI^a^	0.72±0.11	0.59±0.11	<0.001^e^
Umbilical Artery PI^b^	1.03±0.38	0.93±0.24	<0.001^e^
Umbilical Artery S/D^c^	3.46±1.10	2.51±0.57	<0.001^e^
Middle Cerebral Artery RI	0.75±0.17	0.87±0.35	0.014^e^
Middle Cerebral Artery PI	1.45±0.45	1.91±0.86	0.012^e^
RI C/U Index^d^	1.05±0.31	1.59±0.94	<0.001^e^

^a^ RI: resistive index

^b^ PI: palsatility index

^c^ S/D: ratio of systolic to diastolic pressure

^d^ Ratio of cerebral artery RI to umbilical artery RI

^e^ p≤0.05 statistically significant

**Fig. 1:**
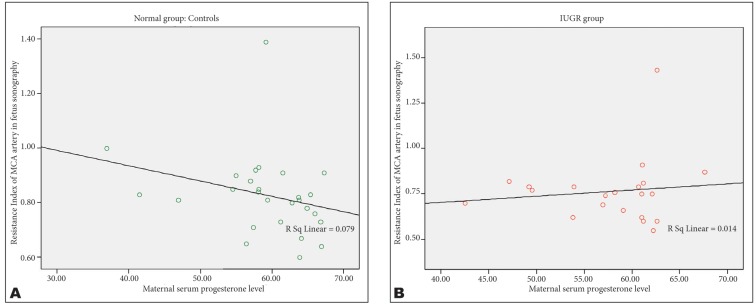
Graphs show the relationship between RI of MCA and progesterone level in normal (A) and IUGR (B) groups.

**Fig. 2:**
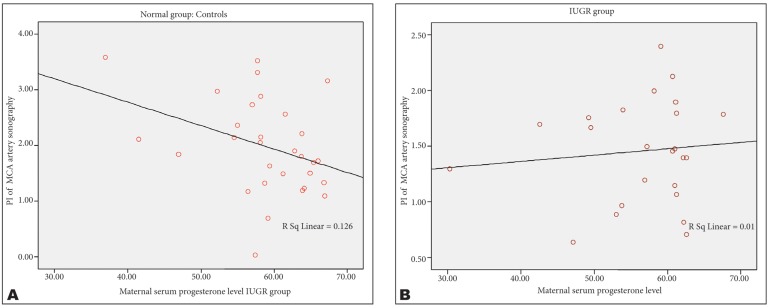
Graphs show the relationship between PI of MCA and progesterone level in normal (A) and IUGR (B) groups.

There were a negative correlation between the maternal progesterone level and middle cerebral RI (r=-0.38, p=0.45) and PI (r=-0.37, p=0.43) in AGA group, but such correlation was not observed in IUGR group (Fig. 1 & Fig. 2) (p=0.95).

There was no correlation between umbilical artery RI, PI, S/D and RI C/U with maternal progesterone in both groups (data not shown).

Using a multiple logistic regression model to remove confounding factors and considering IUGR as the dependent variable, C/U ratio over 1 was detected as the only independent protective factor for IUGR (p=0.003, LR=0.038, CI95%=0.004-0.34). After adjustment for confounding factors such as maternal BMI and gestational age, other variables were not significant predictors for IUGR.

## Discussion

Our results showed that progesterone among IUGR and AGA groups had no significant difference. In the IUGR group, umbilical artery RI, PI and S/D were significantly higher than those in the normal group, and MCA RI and PI were lower than those in the normal group. Also in the IUGR group, RI C/U index was significantly lower than that in the normal group. In the AGA group, there was a negative correlation between progesterone and middle cerebral artery RI and PI. Up to now, investigation about such relationship between progesterone level and Doppler velocimetry indices has not been reported and we could not find a similar study on the internet. This study has assessed this relationship for the first time.

Progesterone is a vasodilator and muscle relaxant and its concentration increases during pregnancy.[13] High levels of progesterone are essential for pregnancy continuity, especially in early pregnancy.[16]

Progesterone induces a rapid vasorelaxant effect on the fetoplacental vasculature, so it can directly influence prostaglandin stimulated smooth muscle contraction.[13]

The vasodilator effect of progesterone may act by various mechanisms. As shown in a previous study, inhibition of nitric oxide reduced the progesterone vasodilatation effect in rabbit’s pulmonary artery.[17]

In one study,[18] it was demonstrated that progesterone and medroxyprogesterone acetate induced prostacyclin synthesis through dose and receptor related pathways in human umbilical venous endothelial cells. Both progesterones increased endothelial prostacyclin by enhancing cyclooxygenase-1 and 2 expression and activity.

In vivo, prostacycline (a potent vasodilator) production by placental cells in IUGR pregnancies was diminished, as compared with normal placental cells. This decrease is due to the loss of synthesis, not due to the increase of metabolism.[19]

Salas et al.[20] showed that in IUGR pregnancies, progesterone as a volume expander decreased after 30 weeks of gestational age. Yanaihara et al.[21] also obtained similar results and reported that progesterone in IUGR pregnancies was lower than that of normal pregnancies.

Ersch et al.[22] reported opposite results, as they observed that 17-Hydroxy progesterone in IUGR infants is higher than normal fetuses at the time of birth. They described this result by chronic stress in IUGR infants and 1-OHP was defined as a marker for NICU (neonatal intensive care unit) admission.

Dawood[23] did not find any significant difference in maternal serum progesterone among IUGR and AGA infants.

Lauring et al.[24] reported that progesterone had no effect on discrimination of IUGR from AGA infants, and there was no correlation between progesterone and neonatal outcome.

Our results confirmed the findings of the two previous studies [23][24] Vasodilator effects of progesterone have been reported in Paonessa et al.,[13] Li et al.[17] and Hermenegildo et al.[18] studies. Considering these reports, we expected to find a relationship between the progesterone level and Doppler velocimetry indices. We expected to see lower progesterone levels in the IUGR group with vascular resistance in the umbilical artery compared with the AGA group without vascular resistance. Despite this, a similarity was detected between progesterone levels of both case and control groups. It seems that progesterone is not a potent vasodilator to cause Doppler velocimetry changes, so it may indicate other pathophysiological mechanisms, such as prostacycline, nitric oxide, and other vasodilators other than progesterone may affect placental resistance.

Our study limitations were the wide gestational age range in the case and control groups, small sample sizes, single measurements and lack of umbilical samples.

The results of this study may be applied for early prediction and treatment of IUGR by measurement of maternal progesterone level and in low level cases it can be added to the treatment regimen. Considering the vasodilator effect of progesterone and vascular resistance in IUGR, we assumed that this resistance may be connected to progesterone level as an etiologic factor and aimed to find such a relationship. Furthermore, we could not find low levels of progesterone in IUGR cases.

However, future studies with larger sample sizes, multiple measurements with specific intervals, umbilical or placental sampling and assessment of perinatal outcome are warranted to confirm the results of the present study.
